# The Potential Role of the NLRP3 Inflammasome as a Link between Mitochondrial Complex I Dysfunction and Inflammation in Bipolar Disorder

**DOI:** 10.1155/2015/408136

**Published:** 2015-05-13

**Authors:** Helena Kyunghee Kim, Wenjun Chen, Ana Cristina Andreazza

**Affiliations:** ^1^Department of Pharmacology and Toxicology, University of Toronto, 1 King's College Circle, Toronto, ON, Canada M5S 1A8; ^2^Department of Psychiatry, University of Toronto, 1 King's College Circle, Toronto, ON, Canada M5S 1A8; ^3^Centre of Addiction and Mental Health, 250 College Street, Toronto, ON, Canada M5T 1R8

## Abstract

Mitochondrial dysfunction and activation of the inflammatory system are two of the most consistently reported findings in bipolar disorder (BD). More specifically, altered levels of inflammatory cytokines and decreased levels of mitochondrial complex I subunits have been found in the brain and periphery of patients with BD, which could lead to increased production of mitochondrial reactive oxygen species (ROS). Recent studies have shown that mitochondrial production of ROS and inflammation may be closely linked through a redox sensor known as nod-like receptor pyrin domain-containing 3 (NLRP3). Upon sensing mitochondrial release of ROS, NLRP3 assembles the NLRP3 inflammasome, which releases caspase 1 to begin the inflammatory cascade. In this review, we discuss the potential role of the NLRP3 inflammasome as a link between complex I dysfunction and inflammation in BD and its therapeutic implications.

## 1. Introduction

Bipolar disorder (BD) is the sixth leading cause of disability worldwide (WHO) with a chronic course, where 25–50% of patients with BD attempt suicide and 50–67% of the patients experience at least one relapse [1]. Despite the urgent need to develop more effective treatments for this disorder, progress has been limited due to a lack of understanding of its pathology.

A growing number of studies are demonstrating mitochondrial dysfunction, especially that of complex I and inflammation in patients with BD [2–7]. For example, a recent review examining microarray findings in BD reported a decrease in complex I subunits that are responsible for transportation of electrons in patients with BD, which could result in increased leakage of electrons and production of reactive oxygen species (ROS) [6]. Altered levels of inflammatory cytokines were also shown in the brain and periphery of patients with BD, including IL-6, TNF*α*, IFN-*γ*, and IL-1*β*, suggesting that activation of the inflammatory system may also play a role in the pathophysiology of BD. Recent studies suggest that mitochondrial production of ROS may be linked to inflammatory activation [8, 9]. In fact, inhibition of complex I and subsequent increase in ROS production lead to increased levels of inflammatory factors such as IL-1*β*, caspase 1, and NF-*κ*B [9, 10].

A potential link between mitochondrial dysfunction and inflammation may be the nod-like receptor pyrin domain-containing 3 (NLRP3) inflammasome, which is a redox sensor that can potentiate the activation of the inflammatory cascade by releasing caspase 1. Indeed, complex I inhibition resulted in the activation of the NLRP3 inflammasome, and decreasing mitochondrial ROS production was able to eliminate inflammasome activation [9, 11]. Therefore, the aim of this review is to explore the link between mitochondrial generation of oxidative stress and inflammation in BD, with a focus on the NLRP3 inflammasome.

## 2. BD and Mitochondrial Dysfunction: A Brief Overview

Mitochondria are energy producing organelles in the cell that generate ATP by transporting electrons through electron transport chain (ETC) complexes I-V. Moreover, they regulate calcium levels and apoptotic processes. Mitochondria are also the main producers of ROS [12]. Complex I, which is responsible for oxidizing NADH and transferring electrons to ubiquinone [13], contains four main subcomplexes: *γ*, *β*, *α*-*λ*, and *λ* that regulate its activity and ROS generation [14]. The *α*-*λ* and *λ* subcomplexes are located on the hydrophilic arm of complex I, which is responsible for electron transfer, and *γ* and *β* subcomplexes are located at the hydrophobic arm, which is responsible for proton pumping [13]. A recent review of microarray studies revealed that patients with BD have decreased mRNA levels of iron-sulfur cluster containing subunits in the hydrophilic portions that are specifically involved in electron transfer, including* NDUFV1*,* NDUFS1*,* NDUFS8*, and* NDUFS7* [6, 15]. On the other hand, the same review revealed that while patients with SCZ have some alterations in mRNA levels of complex I subunits, they do not have alterations in the subunits that are directly involved in the electron transfer process [6, 16, 17]. In support of the microarray findings, decreased protein levels of NDUFS7 and complex I activity were also reported in patients with BD [7, 18]. These findings suggest that patients with BD may be more vulnerable to having increased levels of electron leakage compared to the normal population or patients with SCZ [6]. Leaked electrons from complex I can react with molecular oxygen to produce the superoxide anion, which can escape the mitochondria to undergo a series of reactions to form powerful ROS such as the hydroxyl radical [12]. Oxidative damage to lipids, DNA, and proteins in patients with BD is some of the most consistently reported alterations in BD, which is in agreement with these findings [2, 3, 19].

Superoxide anion and other ROS also play important roles as signaling molecules in the cell through redox sensors that undergo conformational changes, oligomerization, and/or translocation upon detecting ROS or downstream products of ROS release [20]. Nrf2, for example, migrates to the nucleus upon sensing ROS production, and thioredoxin undergoes a structural change upon being modified by ROS [20]. Recent studies have demonstrated that mitochondria may be a potent activator of the immune system through its ability to generate ROS and its interaction with redox sensors in the inflammatory system, such as NLRP3 [8, 9]. These findings suggest that mitochondrial dysfunction in BD may at least be partly responsible for cytokine activation in the central nervous system (CNS) and periphery of patients with BD.

## 3. BD and Inflammation

Alterations in the inflammatory pathway in patients with BD have been reported since 1995, when Maes et al. [21] found increased sIL-6R and sIL-2R levels in patients with mania. Indeed, medical complications related to activation of the inflammatory system such as cardiovascular diseases, diabetes, and obesity are frequently diagnosed in patients with BD [22–24]. Furthermore, patients with BD generally have an earlier onset of cardiovascular diseases [22]. Such findings have inspired the microglial theory, which states that proinflammatory cytokines produced as a result of microglial activation result in disruption of neuroprotective functions, leading to increased vulnerability in BD [23].

Majority of the studies examining inflammation in BD have focused on peripheral samples such as plasma and serum [25–47]. A summary of the findings discussed here can be found in Tables 1, 2, 3, and 4. Multiple studies have reported increased levels of sIL-2R, sIL-6R, TNF-*α*, sTNFR1, IL-1, ILl-12, and TGF-b in BD, while mixed results have been reported for other inflammatory factors, including IL-4, IL-2, IL-8, and IFN-*γ* [25, 27, 30, 32, 38, 40, 42, 44–47]. In this review, we will focus on TNF-*α* and IL-6 for the periphery and the IL-1 pathway for the CNS, as these factors have been consistently reported to be altered in patients with BD.

Despite the large number of studies examining inflammation in BD, there is a lack of agreement regarding the direction of alteration and the cytokines which are altered [23]. However, TNF-*α* and related factors such as sTNFR1 (soluble tumor necrosis factor receptor-1) have been consistently found to be elevated in the periphery of patients with BD [26, 29, 32, 35, 37, 40–42, 44–47]. TNF-*α* is proinflammatory cytokine, which is produced mainly by activated macrophages, CD4+ lymphocytes, and natural killer cells [48, 49]. Upon binding to its receptors, TNFR1 and TNFR2, TNF-*α* can trigger the activation of NF-*κ*B and MAPK pathways [45, 50].

IL-6, which is a proinflammatory cytokine secreted by T cells and macrophages, was also found to be increased in peripheral samples from patients with BD in the majority of studies that were examined in this review. Indeed, IL-6 is one of the cytokines most commonly reported to be altered in BD [29, 31, 32, 40, 42]. IL-6 can mediate fever and acute phase responses. It can also cross the blood brain barrier and trigger the activation of prostaglandin synthesis, which has been implicated in BD [51].

To our knowledge, only three studies have examined inflammation in the CNS in BD [50, 52, 53]. Dean et al. [50] focused on TNF-*α* related factors and pathways in different brain areas (BA24 and BA46) using postmortem brain. Increased concentration of tmTNF-*α* was observed in BA24 for BD, but not in BA46. TNFR2 was found to be decreased in BD subjects [50]. Rao and colleagues [52] focused on the IL-1 pathway and markers of microglial activation using postmortem prefrontal cortex from patients with BD. Higher protein and mRNA levels of IL-1*β*, IL-1R, and MyD88 and microglial/astrocyte markers GFAP and iNOS were found in patients with BD [52]. This was in contrast to Dean et al. [50] who could not detect IL-1*β* in their samples [50]. Findings from Söderlund and others [53] were consistent with Rao et al.'s study [52], showing elevated IL-1*β* levels in the cerebral spinal fluid (CSF) of patients with BD compared to healthy controls. Also, patients who recently had manic or hypomanic episodes showed elevated IL-1*β* levels compared to those who did not [53]. Alterations in cytokine balance in the brain can lead to changes in neurotransmitter levels including dopamine [54, 55], cause microglial activation [56], and activate apoptotic processes [3, 57], all of which have been reported in patients with BD [3, 52, 58].

**Figure 1:**
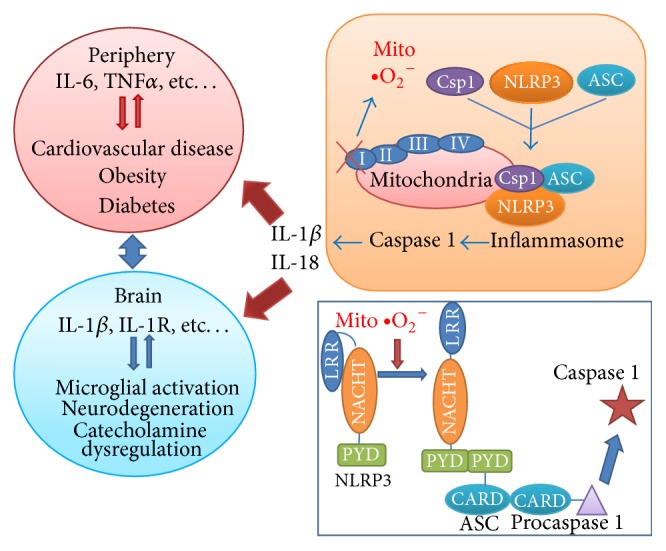
Mitochondrial complex I dysfunction in patients with BD could lead to increased release of superoxide anions, resulting in greater reactive oxygen species (ROS) production. This release of ROS causes a conformational change in NLRP3 such that the pyrin domain (PYD) becomes available recruit ASC. The combining of NLRP3 and ASC that allows for the recruitment of caspase 1 (csp1) through ASC's CARD domain, causing the formation of the NLRP3 inflammasome. The inflammasome then migrates to the mitochondria, allowing it to be close to the site of damage. Activated NLRP3 inflammasome releases caspase 1 into the cytosol, which then cleaves and activates two downstream cytokines, Il-1beta and Il-18, causing them to be released into the extracellular space. These two cytokines cause the activation of downstream pathways, which may differ depending on the type of immune cell. Indeed, NLRP3 inflammasome activation may underlie the different patterns of cytokine activation observed in the brain and peripheral samples of patients with BD, where alterations in cytokines pertaining to the IL-1 pathway have been reported for the brain, while a more general pattern of cytokine activation involving IL-6 and TNF-alpha has been reported in the periphery. Cytokine activation in the periphery can lead to various immune disorders, including cardiovascular disease and diabetes, while, in the brain, it could lead to alterations in neurotransmitters and neurodegeneration.

Interestingly, there has been a lack of agreement between the results found in peripheral samples and the CNS. For example, while TNF-*α* levels are not reported to differ between patients with BD and nonpsychiatric controls in the CNS, its levels are consistently reported to be altered in patients with BD using peripheral samples [29, 32, 35, 37, 40, 42, 47, 50, 52]. Moreover, while increased levels of cytokines in the IL-1 pathway have been reported in the central nervous system, studies examining peripheral samples have not reported alterations in this pathway [23, 40, 42, 47, 52, 53]. The difference between cytokine pathways activated in the periphery and the CNS in BD may be due to the presence of diseases that affect the periphery to a greater extent than the CNS, such as atherosclerosis [24]. The different immune cells that reside in the brain and outside of the blood brain barrier may also be underlying the differences in cytokine profile. On the other hand, the fact that inflammatory activation is found both in the CNS and periphery of patients with BD suggests that the same underlying factor may be causing their activation. Decreased expression of complex I subunits and subsequent generation of mitochondrial ROS may underlie activation of central and peripheral immune cells through the NLRP3 inflammasome [9] (Figure 1).

## 4. The NLRP3 Inflammasome

Recently, studies have shown that oxidative stress and mitochondrial dysfunction have important roles in regulating immune cells of the CNS and the periphery [59]. NLRP3 is a pattern recognition receptor in the inflammatory system that was shown to act as a redox sensor [9]. Cytosolic and membrane-associated pattern recognition receptors can detect danger signals induced by physical and psychological stress [60]. Membrane associated pattern recognition receptors include toll-like receptors that recognize pathogen associated molecular patterns (PAMPs) and damage-associated molecular patterns (DAMPs), which then leads to the release of proinflammatory cytokines such as IL-1*β*, TNF-*α*, and IL-6 [61]. Cytosolic pattern recognition receptors include NOD-like receptors, RIG-like receptors, and DNA sensors [62]. NLRP3 is the most widely studied receptor in the NOD-like receptor family [61]. NLRP3 contains a pyrin domain, a C-terminal leucine-rich domain, and a central nucleotide binding domain [61, 63]. NLRP3 is implicated in a wide variety of inflammatory conditions as it is activated by many different triggers including microbial infection, lipopolysaccharide, tissue damage, ATP, nigericin, and monosodium urate [64, 65]. When NLRP3 is inactive, it resides in the cytoplasm with its leucine-rich domain bound to the central nucleotide binding domain, preventing oligomerization [64]. Upon activation, NLRP3 migrates to the mitochondria associated endoplasmic reticulum membranes and the mitochondria [9]. This was demonstrated by increased colocalization between NLRP3 and mitotracker, which is a fluorescent marker for the mitochondria, and increased levels of NLRP3 in the mitochondrial fraction as well as mitochondria-associated membranes following NLRP3-inflammasome activation [9]. Since ROS are highly reactive and can only travel short distances, it would be ideal for NLRP3 to be localized the mitochondria where ROS is released [9, 66]. However, more studies are needed to test the generalizability of these findings in different systems and in humans. Activation of NLRP3 also causes NLRP3 oligomerization and recruitment of apoptosis-associated speck-like protein containing a CARD (ASC) through pyrin-pyrin domain interaction [9]. Procaspase 1 is also recruited through a CARD-CARD interaction between ASC and procaspase 1, completing the process of NLRP3 inflammasome assembly and activation [63]. NLRP3 inflammasome then releases caspase 1, also known as IL-1*β* converting enzyme. Caspase 1 cleaves pro-IL-1*β* and pro-IL-18 to their mature biologically active forms [67]. IL-1*β* is then released from the cells and binds to the IL-1 type-I receptor, a plasma membrane receptor, and IL-1 receptor-accessory protein to trigger the inflammatory cascade involving downstream signaling molecules such as MYD88 and NF-*κ*B [68]. This leads to increased expression and activation of other inflammatory mediators such as IL-6, TNF-*α*, and prostaglandin E2 [69, 70].

## 5. Mitochondrial Dysfunction and the NLRP3 Inflammasome

Since many different PAMPs and DAMPs can activate NLRP3, it is unlikely that its ligand binding site recognizes all the molecules known to trigger the assembly of the NLRP3 inflammasome [71]. Mitochondrial dysfunction and subsequent production of ROS have received much attention as the common pathway by which different PAMPs and DAMPs trigger inflammasome activation [8, 9, 11, 72]. For example, addition of rotenone, a complex I inhibitor, induces a dose-dependent increase in IL-1*β* secretion [73], while in* Nlrp3* KO mice, the addition of a mitochondrial ETC inhibitor fails to increase IL-1*β* and caspase 1 release [9]. Furthermore, inhibiting liposome-induced mitochondrial ROS release was followed by a decrease in the level of NLRP3-inflammasome activation [11]. While the exact pathway by which mitochondrial ROS leads to NLRP3 inflammasome activation and assembly remains elusive, two possible mechanisms have been proposed: thioredoxin-interacting protein- (TXNIP-) NLRP3 interaction and mitochondrial DNA (mtDNA) release [9, 65].

TXNIP is a tumor suppressor gene and its primary role is to inhibit the redox protein thioredoxin to suppress cell proliferation [9, 74]. Mitochondrial ROS production causes the dissociation between TXNIP and thioredoxin in the mitochondria, causing migration of TXNIP to the cytoplasm, which allows it to directly bind and activate cytoplasmic NLRP3 [74]. Zhou et al. (2011) showed that inflammation stimulating substances such as monosodium urate (MSU), silica, and ATP produce significantly less caspase 1 and IL-1*β* in TXNIP deficient mice, indicating decreased level of NLRP3-inflammasome activation [9]. In addition, in a high glucose concentration environment, islet cells from* Txnip* ^−/−^ and* Nlrp3* ^−/−^ mice showed reduced level of IL-1*β* secretion compared to wild-type mice [9]. TXNIP was also observed to be increased in patients with type II diabetes by a number of different studies [75–77]. Patients with BD are three times more likely to be diagnosed with type II diabetes compared to the general population [78], suggesting that NLRP3 inflammasome activation mediated by TXNIP could be underlying increased peripheral and CNS inflammation in patients with BD.

Another potential mediator between mitochondrial ROS and NLRP3 inflammasome assembly is mtDNA. The role of mtDNA release from the mitochondria to the cytoplasm in NLRP3-inflammasome activation has been suggested following the observation that mtDNA directly binds and activates the NLRP3-inflammasome [10, 79]. Opening of mitochondrial membrane permeability transition pores (MPTs), which allows for mtDNA to escape the mitochondria, is often preceded by mitochondrial ROS production [80]. Also, adding ATP and lipopolysaccharide, which are two well-known stimulators of the NLRP3-inflammasome, increases mitochondrial ROS production and oxidized mtDNA levels in NLRP3 immunoprecipitates [79]. Importantly, it was also found that adding NLRP3 stimuli into cells lacking mtDNA (p0 cells) does not result in IL-1*β* secretion [79], and that the addition of mito-TEMPO, a mitochondrial-ROS scavenger, to bone marrow derived macrophages inhibits IL-1*β* and IL-18 secretion in a dose-dependent manner [81, 82]. Furthermore, preventing the opening of MPTs through the addition of cyclosporine A and thereby preventing mtDNA release inhibit LPS- and ATP-induced IL-1*β* secretion [10].

While the exact mechanism for how mitochondrial dysfunction triggers the assembly of the NLRP3 inflammasome remains to be elucidated, recent studies suggest that release of mitochondrial ROS plays a significant role in this pathway, either through activation of an intermediate redox sensor, such as TXNIP, or by activating apoptotic pathways causing the opening of MPTs [9, 10]. These findings suggest that amelioration of mitochondrial ROS production may aid in decreasing NLRP3-inflammasome activation, which could contribute to decreasing cytokine release in patients with BD.

## 6. Perspectives

With the discovery of immunological alterations in BD, much attention has been given to the possibility of implementing anti-inflammatory agents to treat symptom severity and cognitive decline [24]. An anti-inflammatory drug that was examined in patients with BD is celecoxib, which is a cyclooxygenase-2 inhibitor. Studies performed on rats showed that celecoxib can decrease IL-1*β* concentration in the hypothalamus, prefrontal cortex, and the hippocampus [83, 84]. Celecoxib was also shown to have a significant antidepressant effect in patients with BD, suggesting that anti-inflammatory medications targeting IL-1*β* may be helpful for patients with BD [85]. Aspirin (acetylsalicylic acid), which also inhibits the activity of cyclooxygenase 2 as well as cyclooxygenase 1, is also receiving much attention as a potential treatment option for bipolar depression [86, 87]. Cyclooxygenase enzymes are involved in the arachidonic acid cascade, which can lead to the activation of neuroinflammation pathways [88, 89]. Indeed, low-dose aspirin was found to decrease medication events (change in type of drug, increase in dose, or the number of prescribed drugs) in patients with BD, suggesting that aspirin may aid in stabilizing the symptoms [87].

Since NLRP3 inflammasome is strongly linked to mitochondrial dysfunction and subsequent production of ROS, improving mitochondrial function may contribute to decreasing inflammation in BD. A potential treatment is melatonin, which is a well-established antioxidant and an anti-inflammatory agent that was also demonstrated to target and accumulate in the mitochondria, improve mitochondrial respiration, and inhibit lipopolysaccharide-induced cytokine release [90, 91]. Interestingly, melatonin was found to be decreased in patients with BD, which may underlie disruptions in sleep patterns frequently observed in these patients. These findings suggest that melatonin may aid in decreasing mitochondrial ROS production and subsequent NLRP3 inflammasome activation in BD.

## 7. Conclusion

Mitochondrial complex I dysfunction and chronic inflammation are two of the most consistent findings in BD [2, 3]. Mitochondria are potent activators of the immune system, and this may occur in part through the NLRP3 inflammasome, which is assembled and activated following mitochondrial release of ROS [9]. Since complex I dysfunction in BD could lead to increased production of mitochondrial ROS, NLRP3 inflammasome mediated activation of the inflammatory system may underlie increased cytokine release in the CNS and periphery of patients with BD. Future studies examining the role of the NLRP3 inflammasome in BD will contribute to elucidating the link between two prominent pathophysiological alterations in this disorder, which may reveal pathways that can be used for the development of novel therapeutic interventions that can target both systems to improve symptomatology and cognitive functioning.

## Figures and Tables

**Table 1:** Characteristics of studies examining cytokine alterations in peripheral samples of patients with bipolar disorder.

Author name	Year	Sample size	Sample	Technique used	Cytokines examined
Tsai et al. [25]	2001	31 manic, 31 remission, 31 control	Plasma	ELISA	sIL-2R, sIL-6R

Su et al. [27]	2002	20 BD-I manic, 15 control	Plasma		Stimulated sIL-2R, IL-10, IFN-g

Wadee et al. [28]	2002	45 BD-I manic, 45 control	Serum		CRP

Kim et al. [92]	2002	25 mania, 85 control	Plasma	ELISA	IL-12

Breunis et al. [30]	2003	172 BD I and II, 66 matched control	Serum		sIL-2R

Boufidou et al. [31]	2004	BD I and II, 40 Li treated euthymic, 10 medication naïve, and 20 controls	Plasma	ELISA	Stimulated IL-2, IL-6, IL-10, IFN-g

Kim et al. [93]	2004	70 mania, 96 control	Plasma	ELISA	IFN-g, IL-4, TGF-b1

Liu et al. [94]	2004	29 BD-I manic, 20 controls	Plasma		Stimulated IL-1RA, IL-2, IL-4, IL-10, IFN-g

Knijff et al. [95]	2006	54 BD-I and II, 10 controls	Peripheral blood	FACS	Stimulated IL-2R w/dexamethasone suppression

O'brien et al. [32]	2006	21 control, 12 manic, 9 depressed	Plasma	ELISA	IL-6, IL-8, IL-10, TNF-alpha, sIL-6R

Dickerson et al. [96]	2007	122 BD-I and II, 165 controls	Serum		CRP

Huang and Lin [34]	2007	13 BD-I manic, 23 MDD, 31 controls	Serum		hsCRP

Kim et al. [29]	2007	37 BD-I manic, 74 controls	Plasma	ELISA	Stimulated IL-2, IL-4, IL-10, TNF-a, and IFN-g

Knijff et al. [97]	2007	80 BD-I and II, 59 controls	Serum	ELISA	IL-1b, I-6

Ortiz-Domínguez et al. [42]	2007	33 controls, 20 patients, 10 in manic phase, 10 in depressed phase,	Serum	ELISA	TNF-a, IL-6, IL-1b, IL-2, and IL-4.

Cunha et al. [98]	2008	30 mania, 30 depressed, 20 euthymic, 32 controls	Serum		hsCRP

Kauer-Sant'Anna et al. [35]	2009	60 matched controls, 30 early stage, 30 late stage	Serum	ELISA	BDNF, TNF-a, IL-6 and IL-1

Guloksuz [99]	2010	31 euthymic, 16 control	Serum	Flow cytometry	IL-2, IL-4, IL-5, IL-10, IFN-g, TNF-a

Brietzke and Teixeira [100]	2010	30 euthymic, 30 control	Serum	ELISA	sTNFR1, sTNFR2

Kapczinski et al. [36]	2011	20 manic, 20 depressed, 250 euthymic, 80 control	Serum	ELISA	TNF-a, IL-6, IL-10

Drexhage et al. [47]	2010	38 Euthymic, 22 control	Serum	Flow cytometry, ELISA for sIL-2R	IFN-g, IL-17A, IL-10, IL-6, IL-4, IL-5, IL-8, TNF-a, IL-1b, sIL-2R

Hope et al. [44]	2011	17 “Elevated”, 58 Depressed, 26 Euthymic, 239 control	Plasma	EIA	sTNF-R1, IL1-Ra, IL-6

Barbosa et al. [101]	2011	34 manic, 19 Euthymic, 38 control	Plasma	ELISA	sTNF-R1, IL1-Ra, IL-6

Guloksuz et al. [45]	2012	45 euthymic with subsyndromal symptoms (BD+), 23 without subsyndromal symptoms (BD−), 23 control	Plasma	ELISA	Soluble tumor necrosis factor receptor-1 (sTNF-R1), soluble interleukin-6 receptor (sIL-6R), soluble interleukin-2 receptor (sIL-2R)

Barbosa et al. [37]	2012	25 euthymic, 25 control	Plasma	ELISA	TNF-a, sTNFR1, sTNFR2

Barbosa et al. [37]	2012	30 euthymic, 30 control	Plasma	ELISA	TNF-a, sTNFR1, sTNFR2

Remlinger-Molenda et al. [40]	2012	121 euthymic, 78 control	Serum	cytometry	IL-6, TNF-a, IL-10, IFN-g, IL-2, IL-1b

Cetin et al. [41]	2012	45 euthymic, 23 control	Plasma	ELISA	sTNF-R1, sIL-6R

Tsai et al. [26]	2012	33 manic, 33 remission, 33 control	Plasma	ELISA	IL1-Ra, sTNF-R1

**Table 2:** Characteristics of studies examining inflammatory cytokines in the central nervous system of patients with BD.

Author name	Article year	Sample size	Sample	Technique used	Cytokines examined
Rao et al. [52]	2010	10 BD, 10 control	Postmortem frontal cortex BA 24 and BA 46	Western plot, RT PCR, immunohistochemistry	NMDA receptors, NR-1 and NR-3A, IL-1*β*, IL-1R, MyD88, NF-kB (p50, p65), GFAP, iNOS, c-fos and CD11b, TNF*α*, neuronal nNOS,

Söderlund et al. [53]	2011	BD euthymic patients, r type I (*n* = 15) or type II (*n* = 15)	CSF	An immunoassay-based protein array multiplex system	IL-1b, IL-6,

Dean et al. [50]	2013	10 MDD, 10 BD, 19 SZ, 30 control	Postmortem CNS tissue, BA24 and BA46	Western plot, RT PCR	tmTNF-a, sTNF-a, TNF mRNA, TNFR1, TNFR2, IL-1beta, synaptophysin, PSD95, GFAP43, GFAP41, CD11b and pro-IL1B

**Table 3:** Summary of findings from studies examining alterations in cytokines in peripheral samples from patients with bipolar disorder.

Outcome	*N* studies	Manic vs. controls	Mania vs. euthymia	Depression vs. controls	Euthymia vs. control	Remission vs. control	BD vs. controls	Mania vs. depression	Early stage vs. control	Late stage vs. control	References
sIL-2R	4	+	+	+	+	+	+	+			[25, 27, 30, 45]
sIL-6R	2						+				[25, 41]
IFN-g	6	+−	−			−	−				[29, 31, 40, 93, 94, 99]
TNF-a	11	+		+			+		+	+	[29, 32, 35–37, 40, 42, 47, 99, 101]
sTNFR1	7				+		+				[26, 37, 41, 44, 100, 101]
sTNFR2	6	NS									[37, 100, 101]
TGF-b1	1						+				[93]
IL-1b	4	−						−			[40, 42, 47, 97]
IL-2	6	−		−	−		−				[29, 31, 40, 42, 94, 99]
IL-4	6	+−						+			[29, 42, 47, 93, 94, 99]
IL-5	2										[47, 99]
IL-6	8	+		+	−		−	−			[29, 31, 36, 40, 42, 44, 97]
IL-8	2	+		+							[32, 47]
IL-10	9				−		−				[27, 29, 31, 32, 36, 40, 47, 94, 99]
IL-12	1						+				[92]
IL-1	2						+				[35, 40]
IL-1RA	3	+	+								[26, 44, 94]
CRP	2	+	+	+				+			[28, 96]
hsCRP	2			+							[34, 98]

**Table 4:** Summary of findings from studies examining cytokine alterations in the central nerve system of patients with bipolar disorder.

Outcome	*N* studies	BD vs. controls	References
NR-1 (mRNA and Protein)	1	+	[52]
NR-2A (mRNA and Protein)	1	+	[52]
IL-1*β* (mRNA and Protein)	3	+	[50, 52, 53]
IL-1R (mRNA and Protein)	1	+	[52]
MyD88 (mRNA and Protein)	1	+	[52]
GFAP (mRNA and Protein)	2	+	[50, 52]
iNOS (mRNA and Protein)	1	+	[52]
TNF*α*	2	+	[50, 52]
IL-6	1	−	[53]
tmTNFa	1	+ (at BA 24)	[50]
Astrocyte	1	+	[52]
Microglia markers	1	+	[52]
TNFR2	1	−	[50]
